# Association between spinopelvic parameters and clinical outcomes following hip fracture: an observational retrospective study

**DOI:** 10.3389/fsurg.2026.1734579

**Published:** 2026-02-26

**Authors:** Mehmet Süleyman Abul, Omer Faruk Sevim, Omer Hekim, Mahmut Enes Kayaalp, Engin Eceviz

**Affiliations:** 1 Orthopeadics and Traumatology Department, Kartal Dr. Lutfi Kırdar City Hospital, Istanbul, Türkiye; 2 Orthopeadics and Traumatology Department, Fatih Sultan Mehmet Education and Research Hospital, Istanbul, Türkiye

**Keywords:** femoral neck fracture, pelvic incidence, pelvic tilt, sacral slope, sacral slope difference, spinopelvic parameters

## Abstract

**Background:**

Femoral neck fractures carry a substantial risk of complications such as varus malalignment, avascular necrosis and the need for reoperation. While traditional prognostic factors have been extensively studied, the relevance of postoperative spinopelvic characteristics after internal fixation remains unclear. This study evaluated whether spinopelvic parameters measured on standardized postoperative radiographs are associated with adverse outcomes.

**Methods:**

Ninety-six patients aged 18–60 years who underwent internal fixation for femoral neck fractures were analysed. Demographic variables, fracture characteristics, fixation type and postoperative complications were recorded retrospectively. Sacral slope, pelvic tilt, pelvic incidence and sacral slope difference were measured on lateral lumbosacral radiographs obtained at the 6 month follow-up. Associations with varus deformity, avascular necrosis and reoperation were assessed using univariable and multivariable logistic regression.

**Results:**

Sacral slope difference demonstrated consistent associations with all major complications. Patients with higher sacral slope difference had significantly greater rates of varus deformity, avascular necrosis and reoperation. Higher pelvic tilt was associated with avascular necrosis, and higher pelvic incidence was associated with reoperation. Several multivariable analyses met exploratory criteria due to limited events per variable, and these results should be interpreted with caution. Interobserver reliability for all spinopelvic measurements was excellent.

**Conclusion:**

Spinopelvic parameters, particularly sacral slope difference, were associated with key complications after internal fixation of femoral neck fractures. These postoperative measurements may help identify patients who could benefit from closer follow-up, although they should not be interpreted as predictive factors. Prospective studies are required to validate these associations and clarify their clinical relevance.

## 1 Introduction

Femoral neck fractures are associated with a considerable risk of complications such as varus malalignment, non union and avascular necrosis (1). Although factors including age, fracture displacement, reduction quality and implant stability are known to influence outcomes, these variables alone do not fully explain the heterogeneity observed after internal fixation (2). This has led to increasing interest in whether individual differences in sagittal alignment and pelvic motion may relate to postoperative mechanical and biological behavior of the proximal femur.

Spinopelvic parameters such as sacral slope, pelvic tilt, pelvic incidence and the change in sacral slope between different functional positions describe the interaction between the spine and pelvis during standing and sitting (3). These parameters have gained clinical relevance in hip arthroplasty, where altered lumbopelvic mechanics are associated with instability and non optimal load distribution (4). However, their relevance in patients who undergo internal fixation for femoral neck fractures remains insufficiently explored, and the clinical significance of these parameters in this setting is not well defined.

The relationship between spinopelvic mobility and hip biomechanics provides a rationale for examining these parameters after fracture fixation (5). Daily activities require frequent transitions between standing and sitting, and the resulting changes in pelvic orientation may influence forces transmitted to the femoral neck. Increased or restricted mobility could theoretically alter bending moments and shear forces across the fixation construct. While these biomechanical considerations offer plausible explanations, clinical data evaluating postoperative spinopelvic characteristics in this population are scarce, and causal relationships cannot be inferred.

Given the absence of evidence in the literature, evaluating spinopelvic parameters on standardized postoperative radiographs may offer additional insight into patient specific biomechanical profiles after fixation. These postoperative measurements do not indicate pre-injury alignment but may reflect how the pelvis and lumbar spine function once the patient has resumed daily mobility. Identifying postoperative alignment patterns that correlate with complications may help determine which patients could benefit from closer follow-up. However, these parameters represent postoperative observations and cannot be interpreted as predictive factors.

The aim of this study was to investigate the association between spinopelvic parameters measured on standardized radiographs obtained at postoperative follow-up and the development of complications after internal fixation of femoral neck fractures. We hypothesized that altered spinopelvic mobility, particularly a higher sacral slope difference, would be associated with the occurrence of varus deformity, avascular necrosis and reoperation. The objective of the study was to determine whether these postoperative parameters are associated with outcomes observed during follow-up rather than functioning as predictors of future complications.

## 2 Materials and methods

**Figure 1:**
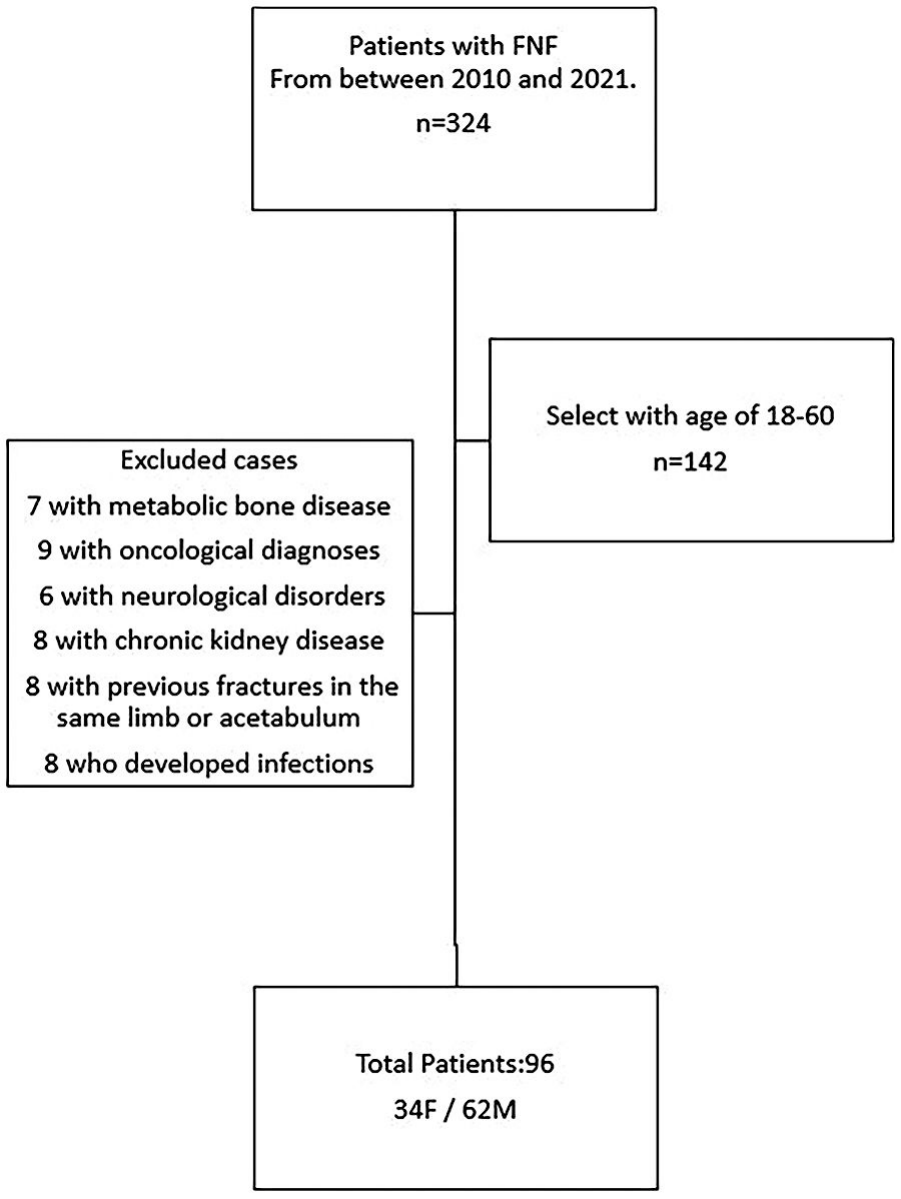
Flowchart illustrating the selection of patients included in the study after applying eligibility and exclusion criteria.

This study was conducted at Kartal Dr. Lutfi Kirdar City Hospital following approval from the Clinical Research Ethics Committee with the research period spanning from November 30, 2022, to February 25, 2023. Designed as a single-center, hospital-based study, the initial retrospective review identified 324 patients who underwent surgical treatment for traumatic femoral neck fractures (FNF) between January 2010 and December 2021. After applying predetermined exclusion criteria, 96 patients aged between 18 and 60 years were eligible for inclusion. Patients older than 60 years were excluded because arthroplasty represents the standard treatment for displaced fractures in this age group, and their inclusion would have introduced heterogeneity in treatment methods. Additional exclusion criteria consisted of metabolic bone disease, oncologic pathology, neurological disorders affecting posture or mobility such as schizophrenia, dementia or Parkinson's disease, chronic kidney disease, ipsilateral previous fractures involving the proximal femur or acetabulum, and postoperative infection. Bone mineral density testing was not routinely performed in this population; therefore, subclinical low bone quality could not be evaluated and remains an unmeasured confounder (Figure 1).

This study utilized a hybrid retrospective prospective design. Clinical variables, fracture characteristics, type of fixation, reduction quality, and postoperative complications were extracted retrospectively from the institutional electronic medical record system. To obtain standardized and reproducible spinopelvic measurements, all included patients were invited for prospective radiographic assessment during their routine 6 month postoperative follow-up visit. Importantly, all spinopelvic parameters analyzed in this study were derived exclusively from these 6 month postoperative radiographs. No preoperative or immediate postoperative spinopelvic images were available, and the study does not include measurements from the acute injury phase. Because all parameters were measured at least 6 months after surgery, they represent postoperative biomechanical alignment and cannot establish temporal or predictive relationships with later complications.

All operations were performed by one of three fellowship trained orthopedic trauma surgeons, each with at least 5 years of independent operative experience. Implant selection between cannulated screws and dynamic hip screw (DHS) constructs generally followed accepted principles related to fracture morphology and anticipated mechanical stability; however, there was no strict institutional algorithm. The final choice was guided by the operating surgeon's preference and intraoperative assessment of stability. All cannulated screw fixations used a standardized three screw configuration. Operative time and intraoperative blood loss were not consistently recorded and therefore were not included in the analysis.

Spinopelvic parameters were measured using standardized lateral lumbosacral radiographs obtained in both standing and sitting positions at the 6 month postoperative follow-up. At this stage of recovery, all patients were able to tolerate standing radiographs, ensuring consistent positioning and reducing feasibility concerns associated with imaging during the acute fracture period. Standing and sitting lateral lumbosacral views were used exclusively to measure sacral slope (SS), pelvic tilt (PT), pelvic incidence (PI), and sacral slope difference (ΔSS). Anteroposterior pelvic radiographs were used solely for fracture classification and implant evaluation, as sagittal alignment cannot be reliably measured on an AP projection.

**Figure 2:**
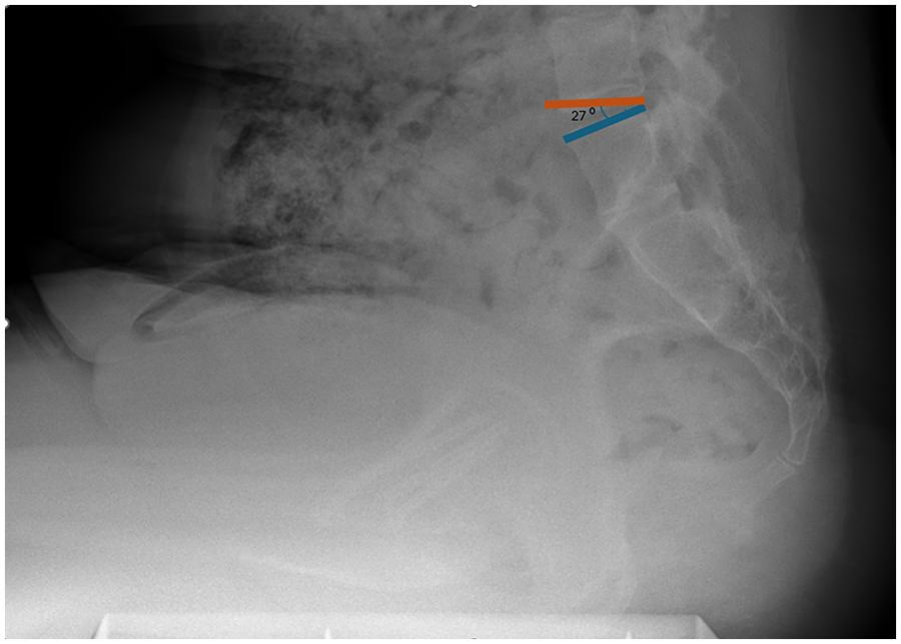
Measurement of sacral slope (SS). The angle between the horizontal reference line and the superior endplate of S1 is identified on the lateral lumbosacral radiograph.

**Figure 3:**
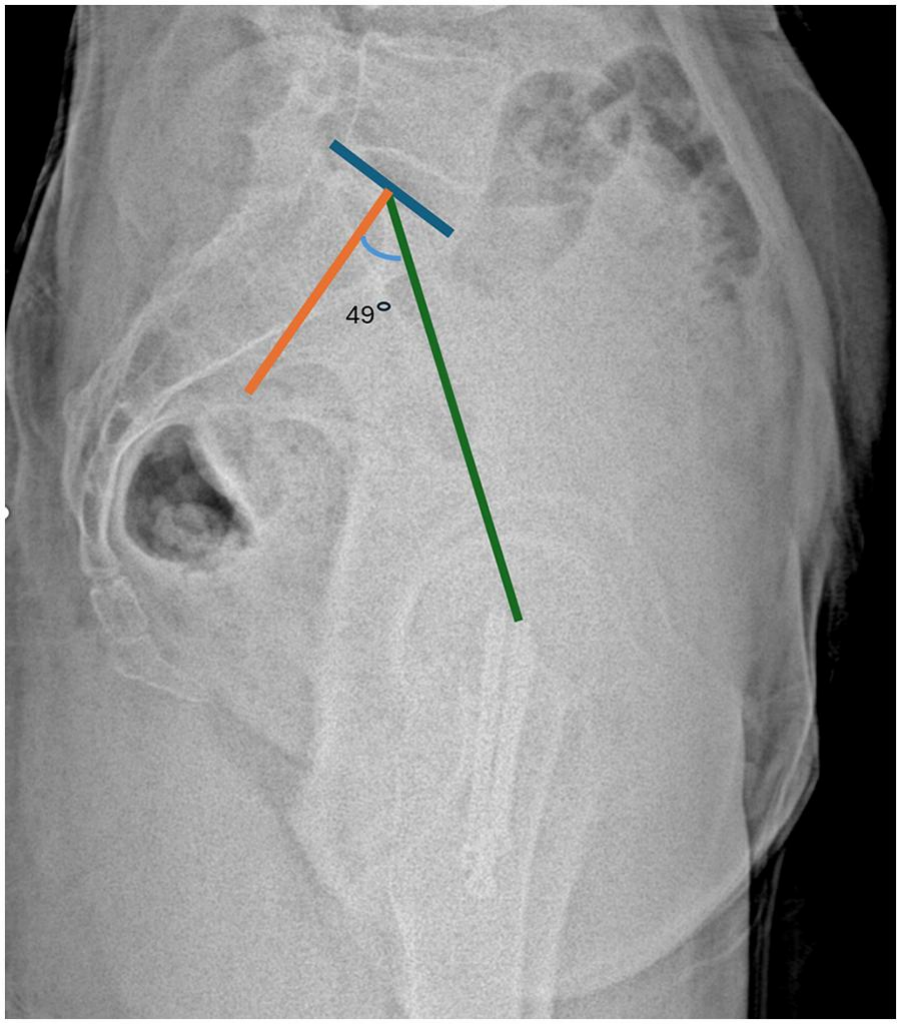
Measurement of pelvic tilt (PT). The angle is formed between a vertical line drawn through the midpoint of the sacral plate and a line connecting this point to the center of the femoral heads.

**Figure 4:**
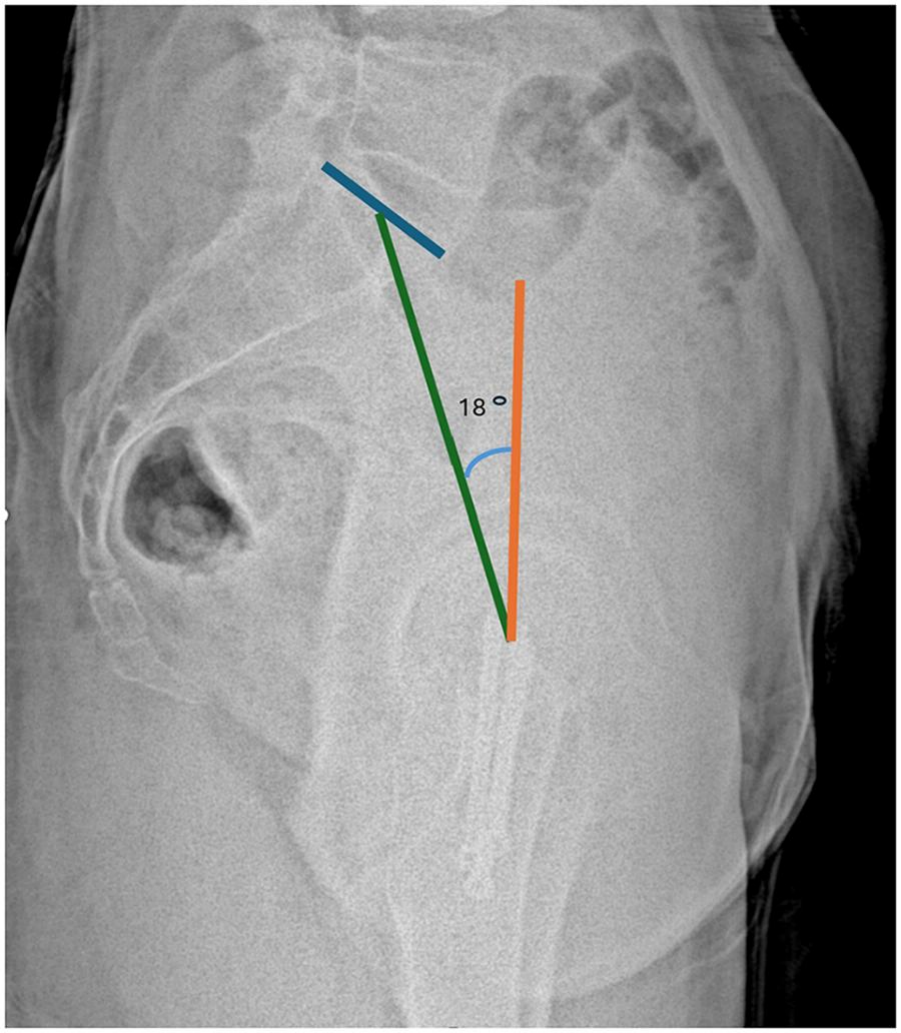
Measurement of pelvic incidence (PI). The angle between a line perpendicular to the S1 endplate and a line connecting the sacral plate midpoint to the femoral head center. PI equals the sum of SS and PT.

**Figure 5:**
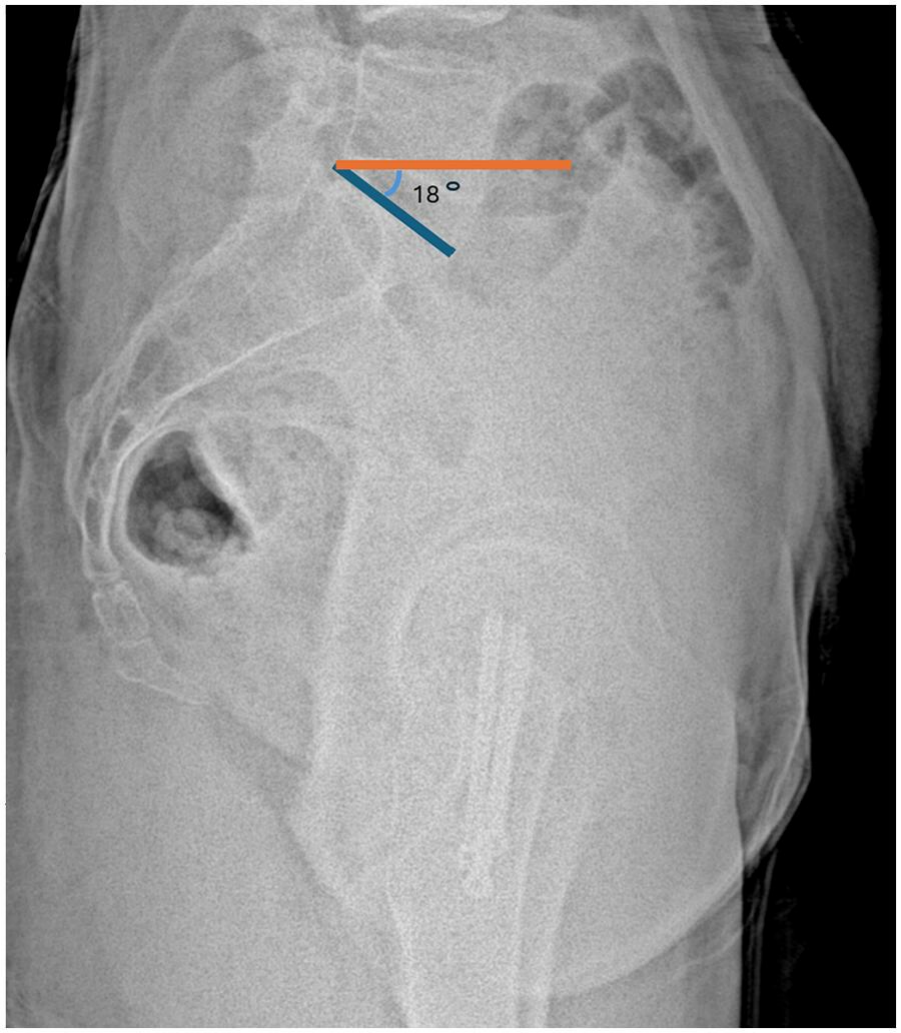
Measurement of sacral slope difference (ΔSS). ΔSS represents the change in sacral slope between standing and sitting lateral lumbosacral radiographs.

Measurements were performed in accordance with established methods. Sacral slope was defined as the angle between the S1 superior endplate and a horizontal reference line (Figure 2). Pelvic tilt was calculated as the angle between a vertical reference through the sacral plate midpoint and the line connecting this point to the femoral head center (Figure 3). Pelvic incidence was defined as the angle between a perpendicular to the S1 endplate and a line joining the sacral plate midpoint with the femoral head center; PI was confirmed mathematically as the sum of SS and PT (Figure 4). Sacral slope difference (ΔSS) represented the absolute difference between SS measured in standing and sitting positions (Figure 5). Postoperative complications, including varus deformity, avascular necrosis (AVN), femoral neck shortening and implant failure, were assessed using radiographs obtained at or after 12 months postoperatively. Varus deformity was defined as loss of more than 10° of neck shaft angle, femoral neck shortening as more than 10 mm, and implant failure as malposition, bending, breakage of screws, or plate breakage. AVN was diagnosed according to radiographic criteria including subchondral sclerosis, subchondral collapse, or femoral head flattening (6, 7).

All radiological measurements were independently performed by two orthopedic surgeons who were blinded to clinical outcomes. Interobserver agreement was quantified by calculating intraclass correlation coefficients using a two way mixed effects model with absolute agreement, with values above 0.90 interpreted as excellent reliability (8).

Collected variables included demographics, body mass index, smoking status, fracture classification according to the AO and Garden systems, energy of trauma, fixation method, and postoperative complications. Due to quasi complete data separation during preliminary modelling, Garden classification was dichotomized into undisplaced (Types I and II) and displaced (Types III and IV) categories for regression analysis (9).

All statistical analyses were performed using SPSS version 25.0. Normality was assessed using the Shapiro–Wilk test and inspection of QQ plots. Continuous variables were presented as mean ± standard deviation or median with interquartile ranges, while categorical variables were presented as frequencies and percentages. Between group comparisons for non normally distributed variables were performed using the Mann–Whitney *U* test. Univariable logistic regression was carried out to examine associations between clinical or radiological variables and postoperative complications including varus deformity, AVN, and reoperation. Implant failure was not modeled due to the very low event count. Variables with a *p*-value below 0.05 in univariable analysis were entered into multivariable logistic regression models using the enter method. To avoid multicollinearity, AO and Garden classifications were not included simultaneously within the same model but were evaluated in separate models. Model adequacy was assessed using the Omnibus Test of Model Coefficients, Nagelkerke R-squared, and the Hosmer-Lemeshow goodness of fit test. Several models had an events per variable ratio below the recommended threshold of 10 and were therefore considered exploratory, with findings interpreted cautiously. Statistical significance was set at *p* < 0.05 for all analyses.

## 3 Results

A total of 96 patients met the inclusion criteria. The mean age was 45 ± 11 years and 34 patients (35.4 percent) were female. The mean body mass index was 25.6 ± 3.6 kg/m^2^. 15 patients (15.6 percent) were smokers. According to the AO classification, 47 patients had 31B1 fractures, 31 had 31B2 fractures and 18 had 31B3 fractures. A high energy trauma mechanism was present in 58 patients (60.4 percent). 72 patients were treated with cannulated screws and 24 with a dynamic hip screw. The reduction quality was graded as good in 79 patients (82.3 percent). The distribution of Garden classification showed that displaced fractures were more common than undisplaced fractures (Table 1). Descriptive values for sacral slope, pelvic tilt, pelvic incidence and sacral slope difference are presented in Table 2.

**Table 1:** Demographic characteristics of patients.

Variables		Patients (*n* = 96)
Age (yr) mean (SD)		45 (11)
Gender (female) *N* (%)		34 (35.4%)
BMI, kg/m^2^ mean (SD)		25.6 (3.6)
Smoking status (*n* yes) *N* (%)		15 (15.6%)
Type of fracture *N* (%)	AO 31B1	47 (49.0%)
	AO 31B2	31 (32.3%)
	AO 31B3	18 (18.8%)
Energy of trauma *N* (%)	High	58 (60.4%)
	Low	38 (39.6%)
Fixation type *N* (%)	Cannulated screw	72 (75.0%)
	DHS	24 (25.0%)
Garden classification *N* (%)	Type 1	6 (6.3%)
	Type 2	35 (36.5%)
	Type 3	28 (29.2%)
	Type 4	27 (28.1%)
Reduction quality *N* (%)	Good	79 (82.3%)
	Bad	17 (17.7%)

Yr, year; BMI, body mass index; SD, standard deviation; DHS, dynamic hip screw.

**Table 2:** Comparison of spinopelvic parameters and complications.

Variables		Sacral slope	Sacral slope difference	Pelvic tilt	Pelvic incidence
Overall		4,818 ± 1.20	11.47 ± 0.78	10.67 ± 0.53	47.33 ± 1.19
Varus	No (*n* = 71)	44 (40–54)	8 (5–14)	8.0 (6.5–14.5)	45 (40–53)
	Yes (*n* = 25)	48 (42–56)	16 (9–20)	10.0 (8.0–14.0)	45 (40–54)
	*p*-value	0.174^a^	**0.003** ^a^	0.404^a^	0.751^a^
AVN	No (*n* = 71)	44 (40–52)	7 (5–12)	8.0 (6.0–13.0)	45 (40–52)
	Yes (*n* = 25)	57 (42–67)	19 (14–23)	14.0 (9.0–15.0)	51 (40–54)
	*p*-value	**0.012** ^a^	**<0.001** ^a^	**0.013** ^a^	0.345^a^
Implant failure	No (*n* = 94)	46 (41–55)	9 (5–17)	9.0 (7.0–14.0)	45 (40–54)
	Yes (*n* = 2)	46 (43–48)	6 (6–7)	11.5 (8.2–14.8)	50 (49–50)
	*p*-value	0.738^a^	0.389^a^	0.990^a^	0.529^a^
Reoperation	No (*n* = 76)	44 (41–53)	8 (5–15)	8.5 (6.8–14.0)	45 (40–52)
	Yes (*n* = 20)	54 (42–58)	16 (11–21)	13.5 (7.8–16.0)	53 (42–59)
	*p*-value	0.078^a^	**0.001** ^a^	0.120^a^	**0.049** ^a^

Median (25th–75th quartile). AVN, avascular necrosis. Overall values are presented as mean ± SD.

Bold values indicate statistical significance (*p* < 0.05).

a Mann–Whitney *U* Test.

When the spinopelvic parameters were compared across outcome groups, sacral slope did not differ significantly between patients with and without varus malalignment or implant failure. However, sacral slope values were significantly higher in patients who developed avascular necrosis (median 57°) compared with those who did not (median 44°). Sacral slope difference showed the most consistent associations. Patients with varus deformity had a median sacral slope difference of 16°, compared with 8° in those without varus. Similarly, patients who developed avascular necrosis had a median difference of 19° vs. 7° among those without necrosis. A higher sacral slope difference was also observed in patients who required reoperation (median 16°) compared with those who did not (median 8°). No statistically significant difference in sacral slope difference was identified between patients with and without implant failure (Table 2). No significant difference was found in trauma to surgery time between patients who developed AVN and those who did not (*p* = 0.745; Supplementary Table S2).

Pelvic tilt did not differ between patients with or without varus malalignment, implant failure or reoperation. However, patients with avascular necrosis demonstrated higher pelvic tilt values at the 6 month postoperative evaluation. Pelvic incidence was not significantly associated with varus deformity or avascular necrosis, but patients who required reoperation had a higher pelvic incidence than those who did not (Table 2).

Univariable logistic regression analyses identified several parameters associated with postoperative complications. For varus deformity, displaced Garden type fractures, AO 31B2 and 31B3 fracture subtypes, poor reduction quality and sacral slope difference were significantly associated with the outcome. Factors associated with avascular necrosis included older age, high energy trauma mechanism, displaced Garden type fractures, AO 31B3 fractures, higher sacral slope, higher sacral slope difference and higher pelvic tilt. Reoperation was associated with trauma mechanism, displaced Garden type fractures, AO 31B2 and 31B3 fracture subtypes, increased sacral slope difference and higher pelvic incidence (Table 3).

**Table 3:** Univariable logistic regression analysis for predictors of postoperative complications.

Predictors	Varus			AVN			Reoperation		
	OR	95% CI	*p*-value	OR	95% CI	*p*-value	OR	95% CI	*p*-value
Age	1.03	0.98–1.08	0.219	**1.05**	**1.00–1.11**	**0.046**	1.04	0.99–1.09	0.143
Gender	0.81	0.31–2.15	0.678	0.81	0.31–2.15	0.678	1.67	0.61–4.55	0.317
BMI (kg/m^2^)	1.11	0.98–1.26	0.116	1.11	0.98–1.26	0.102	1.13	0.98–1.29	0.088
Smoking status	1.53	0.47–5.00	0.486	1.04	0.30–3.62	**0.952**	0.94	0.24–3.72	0.931
Energy of trauma	0.39	0.14–1.08	0.069	**0.14**	**0.04–0.51**	**0.003**	**0.21**	**0.06–0.76**	**0.018**
Garden classification^a^	**4.11**	**1.39–12.17**	**0.011**	**5.71**	**1.78–18.34**	**0.003**	**21.11**	**2.69–165.75**	**0.004**
AO classification									
31B1	1.00 (Ref)			1.00 (Ref)			1.00 (Ref)		
31B2	**4.00**	**1.21–13.21**	**0.023**	2.72	0.91–8.19	**0.075**	**12.38**	**2.51–61.05**	**0.002**
31B3	**10.50**	**2.83–39.03**	**<0.001**	**4.57**	**1.34–15.62**	**0.015**	**14.32**	**2.61–78.70**	**0.002**
Fixation type	0.49	0.15–1.59	0.233	1.24	0.44–3.46	0.687	0.46	0.12–1.74	0.254
Reduction quality	**6.10**	**1.99–18.64**	**0.002**	2.37	0.79–7.12	0.124	2.53	0.80–8.00	0.113
Sacral slope	1.02	0.98–1.06	0.334	**1.05**	**1.01–1.10**	**0.010**	1.04	1.00–1.08	0.079
Sacral slope difference	**1.08**	**1.02–1.15**	**0.010**	**1.21**	**1.12–1.32**	**<0.001**	**1.10**	**1.03–1.17**	**0.005**
Pelvic tilt	1.01	0.93–1.11	0.774	**1.10**	**1.01–1.21**	**0.030**	1.07	0.98–1.18	0.152
Pelvic incidence	1.01	0.98–1.05	0.476	1.03	0.99–1.07	0.150	**1.04**	**1.00–1.09**	**0.041**

AVN, avascular necrosis; BMI, body mass index; OR, odds ratio; CI, confidence interval.

Bold values indicate statistical significance (*p* < 0.05).

a Garden classification recoded: “displaced” (types 3 + 4) vs. “undisplaced” (types 1 + 2) (ref).

Multivariable logistic regression models were constructed separately for each complication type and were limited to variables that met inclusion thresholds in univariable analysis. Sacral slope difference remained a consistent independent variable across models. In the varus model evaluating reduction quality and sacral slope difference together, both remained independently associated with varus alignment at follow-up. In the avascular necrosis models, sacral slope difference remained the only significant parameter, while trauma mechanism and AO 31B3 fracture subtype lost statistical significance. For reoperation, both Garden classification and sacral slope difference maintained significance when entered together in the model. Models with a limited number of EPV were interpreted as exploratory (Table 4). A complete list of all robustness check models is available in Supplementary Table S1.

**Table 4:** Multivariable logistic regression analysis: role of several variables on complications.

Complication and model	Model variables	OR	95% CI	*p*-value
Varus				
Model A1	Reduction quality	**6.12**	**1.90–19.73**	**0.002**
	SS difference	**1.08**	**1.02–1.15**	**0.016**
AVN				
Model B1	Energy of trauma	0.37	0.09–1.65	0.195
	SS difference	**1.19**	**1.09–1.29**	**<0.001**
Model B2^a^	AO, 31B1	Reference		
	AO, 31B2	1.96	0.53–7.25	0.315
	AO, 31B3	3.58	0.82–15.68	0.090
	SS difference	**1.20**	**1.11–1.31**	**<0.001**
Reoperation				
Model C1	Garden classification^b^	**20.04**	**2.48–162.00**	**0.005**
	SS difference	**1.09**	**1.02–1.17**	**0.017**

AVN, avascular necrosis; OR, odds ratio; CI, confidence interval; SS, sacral slope. A complete list of all robustness check models is available in Supplementary Table S1.

Bold values indicate statistical significance (*p* < 0.05).

a MODEL B2 statistical power is limited (event per variable = 8.3; 3 variables/25 events).

b Garden classification recoded: “displaced” (types 3 + 4) vs. “undisplaced” (types 1 + 2) (Ref).

Interobserver reliability for all radiological measurements was excellent, with intraclass correlation coefficients greater than 0.90 for all spinopelvic parameters (Table 5).

**Table 5:** Inter-observer reliability for spinopelvic measurements.

Inter-observer reliability	ICC	95% CI
Sacral slope	0.959	0.90–0.98
Sacral slope difference	0.979	0.92–0.99
Pelvic tilt	0.987	0.90–0.99
Pelvic incidence	0.978	0.95–0.99

ICC, intraclass correlation coefficient; CI, confidence interval. Reliability was calculated using a two-way mixed-effects model with absolute agreement.

## 4 Discussion

The present study evaluated the relationship between postoperative spinopelvic parameters and complications following internal fixation of femoral neck fractures in a young adult population. The main finding was that a greater sacral slope difference, measured on standardized radiographs obtained at 6 months after surgery, was consistently associated with varus malalignment, avascular necrosis and the need for reoperation. In addition, higher pelvic tilt was associated with avascular necrosis, and higher pelvic incidence was associated with reoperation. These findings indicate that postoperative spinopelvic mobility and alignment patterns may reflect biomechanical characteristics relevant to healing after fracture fixation; however, causal or predictive relationships cannot be inferred due to the timing of measurement. Because all spinopelvic parameters were assessed at a fixed 6-month postoperative time point, they should be interpreted as concurrent postoperative biomechanical markers associated with observed complications rather than variables preceding their development.

Sacral slope difference emerged as the most consistently associated parameter across analyses. Patients with greater positional change in sacral slope demonstrated higher rates of varus deformity, avascular necrosis and reoperation. Although the underlying mechanism cannot be determined within this retrospective design, a higher sacral slope difference may reflect alterations in pelvic motion during standing and sitting that influence loading forces across the femoral neck. Increased mobility could expose the fixation construct to greater cyclic stresses, whereas restricted mobility may contribute to asymmetric load transfer (10, 11). These biomechanical interpretations remain speculative but provide potential explanations for the associations observed.

The association between pelvic tilt and avascular necrosis is also notable. Higher postoperative pelvic tilt may reflect a compensatory sagittal posture that shifts the mechanical axis posteriorly, thereby modifying bending forces across the femoral head and neck during gait. Such positional changes may contribute to vascular compromise, although this remains hypothetical and requires further biomechanical validation (12). The finding that higher pelvic incidence was associated with reoperation may similarly indicate that individuals with greater inherent sagittal alignment demands have different postoperative loading behaviour, making them more sensitive to alterations in pelvic mobility after internal fixation (13).

In our cohort, the incidence of avascular necrosis was higher than that reported in large multicenter studies and systematic reviews. This may partly reflect the proportion of high energy trauma mechanisms, which are known risk factors for femoral head necrosis. Additionally, the distinctive postoperative spinopelvic characteristics observed in our population, including greater sacral slope difference and elevated pelvic tilt, may contribute to increased mechanical stress on the femoral head (14). Previous work has suggested an association between pelvic incidence and avascular necrosis after femoral neck fractures, with one study reporting a threshold value above which the risk increases (15). Although spinopelvic factors have not been consistently incorporated into earlier research, our findings support the possibility that postoperative sagittal alignment may be relevant in understanding complication patterns.

Although the literature on spinopelvic parameters in femoral neck fractures is limited, studies in hip arthroplasty (16, 17) and acetabular fracture surgery (18) have demonstrated that sagittal alignment influences joint mechanics, stability and clinical outcomes. Research on the hip spine relationship emphasizes that alterations in pelvic tilt and sacral slope modify femoral version and loading patterns at the hip joint (19). The present findings extend these concepts to patients treated with internal fixation by demonstrating postoperative associations between sagittal pelvic motion and complications such as varus deformity and avascular necrosis. These associations do not imply causation but highlight potential biomechanical relationships worth exploring in future studies.

These results may offer preliminary insight into postoperative assessment strategies. Although the parameters evaluated here were obtained at least 6 months after surgery and therefore cannot function as predictive markers in a temporal sense, postoperative measurements may help identify patients whose biomechanical profiles warrant closer observation. Individuals who demonstrate high sacral slope difference or altered pelvic tilt on postoperative evaluation may benefit from more frequent clinical and radiographic follow-up (12, 20, 21). However, such considerations remain exploratory, and postoperative spinopelvic measurements should not be interpreted as guiding preoperative planning or early rehabilitation protocols. Their potential role is limited to postoperative risk awareness rather than prediction.

The present study has several important limitations. First, bone mineral density was not assessed and represents an unmeasured confounder that may influence healing characteristics and complication risk. Second, although the sample size was adequate for descriptive and univariable analyses, several multivariable models did not meet the recommended events per variable threshold and should therefore be interpreted cautiously. Third, the study population was limited to patients aged 18–60 years, improving internal consistency but limiting generalizability to older adults in whom femoral neck fractures are more common. Although trauma to surgery time did not differ between the avascular necrosis and non-avascular necrosis groups, the limited sample size reduces the ability to definitively exclude an effect of surgical delay. It is also possible that early subclinical avascular changes may have begun before the 6-month radiographic assessment, particularly in patients who later developed avascular necrosis, which precludes any inference regarding temporal directionality or causality. Finally, detailed assessment of lumbar spinal morphology was not feasible because dedicated full spine imaging was not routinely obtained.

Despite these limitations, this study provides novel data on the relationship between postoperative spinopelvic characteristics and complications after internal fixation of femoral neck fractures. The findings suggest that sacral slope difference, pelvic tilt and pelvic incidence, when measured in the postoperative period, may serve as markers reflecting biomechanical behaviour associated with healing. Future large scale prospective studies incorporating longitudinal imaging, assessment of bone quality, and comprehensive evaluation of spinopelvic dynamics are needed to validate these associations and clarify their clinical relevance.

## 5 Conclusion

Spinopelvic parameters, particularly sacral slope difference, were associated with varus malalignment, avascular necrosis and reoperation after internal fixation of femoral neck fractures. These findings indicate that postoperative spinopelvic mobility may reflect biomechanical patterns observed in patients who later develop complications and may help inform which individuals could benefit from closer clinical and radiographic follow-up. Further prospective studies are required to determine the temporal and clinical significance of these associations. These findings should be interpreted in the context of a younger adult population and may not be directly generalizable to older patients with femoral neck fractures.

## Data Availability

The raw data supporting the conclusions of this article will be made available by the authors, without undue reservation.
