# Supplementary material for: Association between spinopelvic parameters and clinical outcomes following hip fracture: an observational retrospective study
Source: Front Surg. 2026 Feb 26;13:1734579. doi: 10.3389/fsurg.2026.1734579 (PMC12979497; doi:10.3389/fsurg.2026.1734579)
Supplement: Supplementary file 1 [file Table1.docx]

**Supplementary Table 1:** Logistic regression analysis: role of several variables on complications

| **Complication & Model** | **Model Variables** | **OR** | **95% CI** | **p-value** |
| --- | --- | --- | --- | --- |
| **VARUS** | | | | |
| **MODEL A1** | **Reduction Quality** | **6.12** | **1.90–19.73** | **0.002** |
|  | **SS Difference** | **1.08** | **1.02–1.15** | **0.016** |
| ****MODEL A2** | AO, 31B1 | Reference | | |
|  | **AO, 31B2** | **3.42** | **1.01–11.63** | **0.049** |
|  | **AO, 31B3** | **9.18** | **2.39–35.22** | **0.001** |
|  | **SS Difference** | **1.07** | **1.00–1.14** | **0.045** |
| **MODEL A3** | ***Garden Classification** | **3.71** | **1.22–11.27** | **0.021** |
|  | **SS Difference** | **1.07** | **1.01–1.14** | **0.024** |
| **AVN** | | | | |
| **MODEL B1** | Energy of Trauma | 0.37 | 0.09–1.65 | 0.195 |
|  | **SS Difference** | **1.19** | **1.09–1.29** | **<0.001** |
| ****MODEL B2** | AO, 31B1 | Reference | | |
|  | AO, 31B2 | 1.96 | 0.53–7.25 | 0.315 |
|  | AO, 31B3 | 3.58 | 0.82–15.68 | 0.090 |
|  | **SS Difference** | **1.20** | **1.11–1.31** | **<0.001** |
| **MODEL B3** | ***Garden Classification** | **6.83** | **1.64–28.45** | **0.008** |
|  | **SS Difference** | **1.22** | **1.11–1.33** | **<0.001** |
| ****MODEL B4** | Sacral Slope | 0.99 | 0.94–1.05 | 0.826 |
|  | **SS Difference** | **1.21** | **1.10–1.33** | **<0.001** |
|  | Pelvic Tilt | 1.03 | 0.91–1.16 | 0.648 |
| **MODEL B5** | Age | 0.99 | 0.93–1.06 | 0.795 |
|  | **SS Difference** | **1.22** | **1.11–1.34** | **<0.001** |
| **REOPERATION** | | | | |
| **MODEL C1** | ***Garden Classification** | **20.04** | **2.48–162.00** | **0.005** |
|  | **SS Difference** | **1.09** | **1.02–1.17** | **0.017** |
| **MODEL C2** | Energy of Trauma | 0.34 | 0.08–1.37 | 0.129 |
|  | SS Difference | 1.07 | 1.00–1.15 | 0.055 |
| **MODEL C3** | **SS Difference** | **1.09** | **1.02–1.16** | **0.015** |
|  | Pelvic Incidence | 1.03 | 0.99–1.08 | 0.144 |

AVN: Avascular necrosis; OR: Odds Ratio; CI: Confidence Interval; SS: Sacral Slope. *Garden Classification recoded: 'Displaced' (Types 3+4) vs. 'Undisplaced' (Types 1+2) (Ref). **MODEL A2, B2 and B4 statistical power is limited (Event Per Variable = 8.3; 3 variables / 25 events).

**Supplementary Table 2:** Comparison of trauma-to-surgery time between AVN and non-AVN groups

| Group | N | Median (IQR) | Mean Rank | p-value |
| --- | --- | --- | --- | --- |
| No AVN | 71 | 8 (6–12) | 47.96 | **0.745** |
| AVN | 25 | 8 (6–12) | 50.02 |  |

Mann-Whitney U Test
